# Supplementary material for: Predicting maths anxiety from mathematical achievement across the transition from primary to secondary education
Source: R Soc Open Sci. 2019 Nov 27;6(11):191459. doi: 10.1098/rsos.191459 (PMC6894589; doi:10.1098/rsos.191459)
Supplement: Supplementary Information for Predicting Maths Anxiety From Mathematical Achievement Across the Transition From Primary- to Secondary-Education [file rsos191459supp1.pdf]

ROYAL SOCIETY OPEN SCIENCE

rsos.royalsocietypublishing.org

Research

Check for updates

Article submitted to journal

Subject Areas:
Maths anxiety, School Transition, Anxiety symptoms

Keywords:
Maths, Anxiety, Education

Author for correspondence:
Andy P. Field
e-mail: andyf@sussex.ac.uk

Supplementary Information for Predicting Maths Anxiety From Mathematical Achievement Across the Transition From Primary- to Secondary-Education

Andy P. Field[1], Danielle Evans[1], Tomasz Bloniewski[2,3], Yulia Kovas[2,3]

[1]School of Psychology, University of Sussex, UK
[2]Department of Psychology, Goldsmiths, University of London, UK
[3]International Centre for Research in Human Development, Tomsk State University, Russia.

Table 1 shows the pattern of missing data for predictors of maths anxiety. Table 2 shows the main model from the paper (Table 4 in the main paper) but conducted on standardized scores. The parameter estimates in this table are, therefore, standardized coefficients. Similarly, Table 3 shows the *post hoc* analysis from the paper (Table 5 in the main paper) but conducted on standardized scores.

THE ROYAL SOCIETY PUBLISHING

© 2014 The Authors. Published by the Royal Society under the terms of the Creative Commons Attribution License http://creativecommons.org/licenses/by/4.0/, which permits unrestricted use, provided the original author and source are credited.

rsos.royalsocietypublishing.org R. Soc. open sci. 000000

Table 1. Missing data patterns for predictors of maths anxiety

	Missing value pattern						
Predictor	1	2	3	4	5	6	7
Biological sex	1	1	1	1	1	1	1
Maths (pre-transition)	1	1	1	1	1	1	1
Maths (change)	1	1	1	1	1	1	1
SES	1	1	0	0	1	1	0
SDQ (pre-transition)	1	1	1	1	0	0	0
SDQ (change)	1	1	1	1	0	0	0
Verbal attainment	1	0	1	0	1	0	0
General anxiety	1	1	1	1	1	1	1
n	952	82	36	3	12	17	2

Table 2. Version of Table 4 from the paper but with the model fitted to standardized scores

Predictor	b	95% CI	t	DF	p
Intercept	0.13	[0.06, 0.20]	3.92	1,091.38	0.00
SES	0.04	[−0.02, 0.10]	1.45	999.48	0.15
Biological sex	-0.37	[−0.48, −0.26]	-6.44	1,090.65	0.00
SDQ (pre-transition)	0.13	[0.06, 0.20]	3.51	1,002.37	0.00
SDQ (change)	0.12	[0.06, 0.19]	3.54	1,024.77	0.00
Verbal attainment	-0.01	[−0.08, 0.05]	-0.46	836.27	0.65
Maths (pre-transition)	-0.24	[−0.30, −0.18]	-8.03	1,073.05	0.00
Maths (change)	-0.05	[−0.11, 0.00]	-1.95	1,091.17	0.05
General anxiety	0.26	[0.21, 0.32]	9.41	1,088.57	0.00

Authors' Contributions. AF produced this supplementary information.

Table 3. Key model parameters for predictors of maths anxiety at age 18 (exploratory model)

Predictor	b	95% CI	t	DF	p
Intercept	0.13	$[0.06, 0.19]$	3.76	1,087.31	0.00
SES	0.04	$[-0.02, 0.10]$	1.43	993.89	0.15
Biological sex	-0.38	$[-0.49, -0.27]$	-6.62	1,086.47	0.00
SDQ (pre-transition)	0.15	$[0.07, 0.24]$	3.58	991.69	0.00
SDQ (change)	0.14	$[0.06, 0.22]$	3.33	1,000.24	0.00
Verbal attainment	-0.01	$[-0.08, 0.05]$	-0.44	844.38	0.66
Maths (pre-transition)	-0.27	$[-0.35, -0.20]$	-7.13	1,075.47	0.00
Maths (change)	-0.07	$[-0.14, -0.00]$	-2.07	1,087.59	0.04
General anxiety	0.26	$[0.21, 0.32]$	9.42	1,084.05	0.00
Sex Œ Maths (pre-transition)	0.08	$[-0.02, 0.19]$	1.51	1,088.23	0.13
Sex Œ Maths (change)	0.06	$[-0.05, 0.16]$	1.01	1,088.08	0.31
Sex Œ SDQ (pre-transition)	-0.10	$[-0.24, 0.05]$	-1.32	1,071.54	0.19
Sex Œ SDQ (change)	-0.06	$[-0.20, 0.08]$	-0.84	1,061.31	0.40
